# Putting a finishing touch on GECIs

**DOI:** 10.3389/fnmol.2014.00088

**Published:** 2014-11-18

**Authors:** Tobias Rose, Pieter M. Goltstein, Ruben Portugues, Oliver Griesbeck

**Affiliations:** Max-Planck-Institute of NeurobiologyMartinsried, Germany

**Keywords:** buffering, calcium, fluorescent protein, FRET, imaging, neuronal activity, segmentation

## Abstract

More than a decade ago genetically encoded calcium indicators (GECIs) entered the stage as new promising tools to image calcium dynamics and neuronal activity in living tissues and designated cell types *in vivo*. From a variety of initial designs two have emerged as promising prototypes for further optimization: FRET (Förster Resonance Energy Transfer)-based sensors and single fluorophore sensors of the GCaMP family. Recent efforts in structural analysis, engineering and screening have broken important performance thresholds in the latest generation for both classes. While these improvements have made GECIs a powerful means to perform physiology in living animals, a number of other aspects of sensor function deserve attention. These aspects include indicator linearity, toxicity and slow response kinetics. Furthermore creating high performance sensors with optically more favorable emission in red or infrared wavelengths as well as new stably or conditionally GECI-expressing animal lines are on the wish list. When the remaining issues are solved, imaging of GECIs will finally have crossed the last milestone, evolving from an initial promise into a fully matured technology.

## INTRODUCTION

Genetically encoded calcium indicators (GECIs) have come of age. Since the first demonstration of FRET (Förster Resonance Energy Transfer)-based prototypical sensors such as the Cameleons (Miyawaki et al., 1997, 1999) and the first single fluorophore calcium sensors (Baird et al., 1999), these two major classes have evolved high performance variants in which signal strength was optimized in iterative steps of improvements and validation. Among FRET based sensors Cameleons, which exploit the interaction of Calmodulin with the binding peptide M13 as a calcium sensing mechanism, saw several rounds of improvements of their signal strength (Nagai et al., 2004; Horikawa et al., 2010). Troponin C has been used as a more biocompatible alternative to Calmodulin in FRET sensors (Heim and Griesbeck, 2004). These sensors also underwent several rounds of engineering (Mank et al., 2006, 2008). Among single fluorophore sensors GCaMP type sensors (Nakai et al., 2001) became the most popular class, chosen from several initial architectures. Variants with ever increasing sensitivity to neuronal activity were generated (Ohkura et al., 2005, 2012a; Tian et al., 2009; Akerboom et al., 2012), as were blue and red emitting color variants (Zhao et al., 2011; Akerboom et al., 2012; Ohkura et al., 2012b). Finally, large scale mutagenesis and screening approaches have resulted in GECIs that match or even exceed the *in vivo* sensitivity of the synthetic calcium dye OGB-1, often referred to as a standard against which response properties of new GECIs were compared to (Chen et al., 2013; Thestrup et al., 2014).

Genetically encoded calcium indicators finally made it possible to label specific types of neurons *in vivo* and even allowed targeting to subcellular compartments and repeated imaging of identified neurons over long periods of time. For several small genetically tractable organisms with strong body walls or cuticulae such as in *Caenorhabditis elegans* or *Drosophila*, which made access and loading of dyes from the outside challenging, expression of GECIs was the only feasible way to image neuronal activity. In many aspects imaging of GECIs has thus become a well-established technology that enables experiments that previously were not possible.

What is the ideal GECI for imaging neuronal physiology? Obviously this will depend on the experimental situation and the neuronal cell types to be imaged. Nevertheless, a number of general criteria may be derived that a GECI should strive to include in the ideal case. (i) It should be bright enough to identify expressing cells even at rest, allow an estimate of the amount of indicator expressed in a given cell after gene transfer, and possibly reveal fine details of its architecture. (ii) It should be readily expressed at sufficient levels by the standard methods for gene transfer and transgenesis. (iii) It should exhibit a linear relationship between the changes in free calcium and the fluorescence change of the indicator. (iv) For reporting neuronal activity it should be sensitive enough to faithfully report small calcium elevations due to firing of single action potentials (APs) in single trials *in vivo*, ideally at lower magnification and faster scanning rate to sample large numbers of neurons. (v) It should not perturb cells that express the indicator by buffering of physiological calcium or other unwanted biological side effects. (vi) It should minimize artifacts due to specimen movement, photobleaching, or other perturbing causes. (vii) Finally, it should have sufficiently fast binding kinetics to accurately follow calcium fluctuations, if it is used as a reporter of neuronal activity.

In view of these criteria we will discuss some of the current issues in quantifying neuronal signals with GECIs and point to some further desirable improvements to finally turn imaging of GECIs it into a mature, fully fledged technology for the study of neuronal function.

## QUANTIFYING NEURONAL ACTIVITY WITH GECIs – DEALING WITH NON-LINEARITY

**FIGURE 1:**
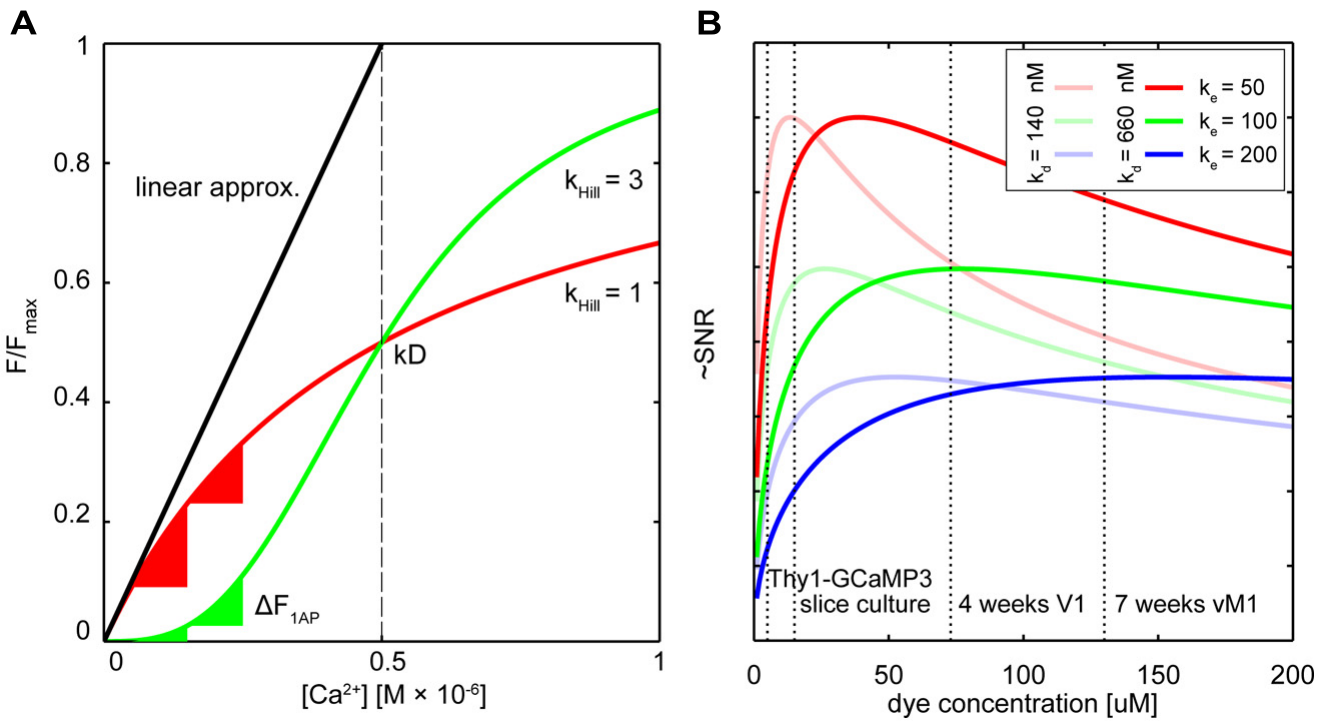
**Dealing with non-linearity and buffering. (A)** Relative fluorescence changes of two hypothetical calcium indicators with similar affinity but different cooperativity (OGB-like: red trace, Hill slope (*K*_hill_) = 1, GECI-like: green trace *K*_hill_ = 3) in response to varying calcium concentrations. The black line shows the commonly used linear calibration function (Eq. 1). Only for small changes in Ca^2+^ – well below the indicator *K*_d_ – this function describes the fluorescence response of the more linear indicator (red trace) well. Even though the non-linear indicator (green trace) responds approximately linear at intermediate Ca^2+^ levels around the *K*_d_, the linear calibration function is left-shifted and therefore prominently underestimates the actual Ca^2+^ concentration from the measured fluorescence. Note that the same absolute change in Ca^2+^ starting from different resting calcium levels will lead to very different fluorescence changes of the non-linear indicator (green triangles) whereas the linear indicator responds with comparable changes (red triangles). **(B)** Hypothetical relation between added exogenous buffer capacity (i.e., increasing indicator concentrations) and signal-to-noise ratio (SNR; Göbel and Helmchen, 2007). Shown are different endogenous buffer capacities corresponding roughly to the range of reported values for pyramidal and subclasses of inhibitory neurons (red: κ_e_ = 50; green κ_e_ = 100; blue κ_e_ = 200; assumed resting [Ca^2+^]_i_ 50 nM). Two hypothetical indicators with different affinities similar to reported *in vitro* values for GCaMP3 and GCaMP6s are shown [bold lines: *K*_d_ = 660 nM, faded lines *K*_d_ = 140 nM; true *in vivo K*_d_ of genetically encoded calcium indicators (GECIs) may vary]. Note that SNR optimal indicator concentrations would be reached at ∼25–50 μM. Here, ‘balanced loading’ is achieved where endogenous and exogenous buffer capacities are equal. Dashed vertical lines indicate four reported *in situ* indicator concentrations using different gene delivery methods and expression times for the GECI GCamP3: Emx1-Cre:Ai38 mice, 5.4 μM (Zariwala et al., 2012); hippocampal slice culture after single-cell electroporation, 15 μM (Huber et al., 2012); primary visual cortex after 4 weeks of viral (AAV) infection, 72 μM (Zariwala et al., 2012); 7 weeks after AAV infection in vibrissal motor cortex (Huber et al., 2012), 130 μM.

The biochemical and optical properties of the latest generation of GECIs rival and in some aspects even surpass those of synthetic calcium indicators (Chen et al., 2013; **Table 1**). It is now possible to detect the somatic calcium influx associated with individual APs with high reliability *in vivo*. Even calcium signals following synaptic activation can now be monitored chronically in live animals (Chen et al., 2013). Yet, in one point most GECIs are clearly inferior to their synthetic counterparts: linearity with respect to the actual calcium concentration. Indicator non-linearity renders the direct deduction of absolute changes in calcium from the relative changes in fluorescence challenging. Robust quantification with the commonly used calibration methods is only possible in the ‘linear’ regime of a calcium indicator (Grynkiewicz et al., 1985; Neher, 1995; Maravall et al., 2000; Yasuda et al., 2004), well below its *K*_d_ value (**Figure 1A**). Only in this range the fluorescence intensity (or fluorescence ratio) change Δ*F/F* or *ΔR/R* of the indicator is approximately proportional to the cellular Ca^2+^ concentration ([Ca^2+^]_i_). Most synthetic indicators with linear response curves (Hill coefficient ∼1) show a simple saturation function of Δ*F/F or* Δ*R/R* vs. [Ca^2+^]. The saturation fluorescence *F*_max_ in response to [Ca^2+^] > > K_d_ is used together with the indicator fluorescence *F_min_* at zero [Ca^2+^] to calibrate the fluorescence response:

**Table 1:** Comparison of current generation genetically encoded calcium indicators (GECIs) for *in vivo* usage with OGB.

Indicator	Fluorophore(s)	Ca^2+^ sensing domain	*in vitro K*_D_ (nM)	Hill slope	Rise (s)	Decay(s)	Single AP(Δ*F*/*F* or Δ*R*/*R*)	Description	Reference
***Synthetic***
OGB	Oregon green	BAPTA	260	1.48	0.24^a^ 0.09^b^	0.38^a^ 2.11^b^	10.0 ± 0.9%^j^ 5.2 ± 0.9%^k^	High linearity; high baseline brightness; fast kinetics; acute usage (<12 h)	Kerr et al. (2005), Hendel et al. (2008), Tada et al. (2014)
Cal-520	–	BAPTA	320	–	0.06^b^	0.69^b^	18.8 ± 0.8%^k^	Latest generation synthetic indicator	Tada et al. (2014)

***Single FP***
GCaMP3	cpEGFP	Calmodulin	345–660	2.1–2.5	0.08^c^	0.61^c^ 0.64^d^	7.9 ± 2.8%^j^ 17.4 ± 3.5%^n^	–	Tian et al. (2009), Akerboom et al. (2012), Chen et al. (2013)
GCaMP5G	cpEGFP	Calmodulin	450–460	2.5	0.15^e^	0.61^e^	–	–	Akerboom et al. (2012), Chen et al. (2013)
GCaMP5K	cpEGFP	Calmodulin	189	3.8	0.06^f^	0.27^f^	3.6 ± 1.9%^l^	High affinity; High non-linearity	Akerboom et al. (2012), Chen et al. (2013)
GCaMP6	cpEGFP	Calmodulin	158	–	–	0.46^d^	27.9 ± 4.5%^n^	–	Ohkura et al. (2012b)
GCaMP8	cpEGFP	Calmodulin	200	–	–	0.43^d^	37.8 ± 5.2%^n^	–	Ohkura et al. (2012b)
GCaMP6f	cpEGFP	Calmodulin	375	2.27	0.14^e^ 0.05^f^	0.38^e^ 0.14^f^	19 ± 2.8%^l^	Medium–high affinity; low baseline brightness; faster kinetics	Chen et al. (2013)
GCaMP6m	cpEGFP	Calmodulin	167	2.96	0.14^e^ 0.08^f^	0.87^e^ 0.27^f^	13 ± 0.9%^l^	High affinity; low baseline brightness; intermediate kinetics	Chen et al. (2013)
GCaMP6s	cpEGFP	Calmodulin	144	2.90	0.16^e^ 0.18^f^	1.14^e^ 0.55^f^	23 ± 3.2%^l^	High affinity; low baseline brightness; slower kinetics	Chen et al. (2013)
***FRET***
YC3.60	ECFP/cpVenus	Calmodulin	250	1.7	0.82^g^	0.73^g^	2.0 ± 0.09%^k^ 5.5 ± 1.2%^m^	–	Nagai et al. (2004), Hendel et al. (2008), Horikawa et al. (2010), Lütcke et al. (2010)
YC-Nano15	ECFP/cpVenus	Calmodulin	15.8	3.1	–	∼ 4^h^	10.4 ± 1.9%^m^	High affinity; high baseline brightness; slower kinetics	Horikawa et al. (2010), Thestrup et al. (2014)
TN-XXL	ECFP/cpCitrine	Troponin	800	1.5	1.04^g^	0.88^g^	1.6 ± 0.3%^n^	–	Mank et al. (2008), Thestrup et al. (2014)
Twitch2B	mCerulean3/ cpVenus	Troponin	200	1.31	–	2.11^i^	26.5 ± 3.8%^o^	High linearity; high baseline brightness; slower kinetics	Thestrup et al. (2014)
Twitch3	ECFP/cpCitrine	Troponin	250	1.42	–	2.05^i^	5.7 ± 0.7%^p^	High linearity; high baseline brightness; slower kinetics	Thestrup et al. (2014)

*Overview of parameters describing the function of the commonly used GECIs, their predecessors and the ‘golden standard’ OGB.*

*K_D_ measured by stop-flow measurement using purified protein.*

*Rise and decay:*

*^a^Single exponential fit (τ_rise_, τ_decay_), 40 APs (20 Hz) in *drosophila* larval neuromuscular junction (NMJ) Hendel et al. (2008).*

*^b^Rise time (10-90), decay time constant from double exponential fit to single AP in acute brain slices of mouse barrel cortex Tada et al. (2014).*

*^c^Half-rise time (t_1/2_), half-decay time (t_1/2_) for 10 APs (83Hz) in hippocampal slice culture Tian et al. (2009).*

*^d^Single exponential fit (decay τ_1/2_), 1 AP in hippocampal slice culture Ohkura et al. (2012b).*

*^e^Full-rise time, half-decay time (t_1/2_), 20 Hz in *drosophila* larval NMJ Chen et al. (2013).*

*^f^Full-rise time, half-decay time (t_1/2_) for single AP induced signals in mouse V1 *in vivo*Akerboom et al. (2012) or Chen et al. (2013).*

*^g^Single exponential fit (τ_rise_, τ_decay_), 80 APs (40 Hz) in *drosophila* larval NMJ Hendel et al. (2008) or Mank et al. (2008).*

*^h^Single exponential fit (τ_decay_), for 10 APs (20 Hz) in mouse brain Horikawa et al. (2010).*

*^i^Single exponential fit (τ_decay_), for 10 APs (83 Hz) in dissociated hippocampal culture Thestrup et al. (2014).*

*Single AP Δ*F*/*F* or Δ*R*/*R* measured in:*

*^j^Mouse M1 or S1 *in vivo*Kerr et al. (2005) or Tian et al. (2009).*

*^k^Mouse barrel cortex *in vivo*Lütcke et al. (2010) and Tada et al. (2014).*

*^l^Mouse V1 *in vivo*Chen et al. (2013).*

*^m^Mouse cortex, acute brain slices Lütcke et al. (2010) or Horikawa et al. (2010).*

*^n^Hippocampal slice culture Mank et al. (2008) and Ohkura et al. (2012b).*

*^o^Acute cortical slice Thestrup et al. (2014).*

*^p^Dissociated hippocampal culture Thestrup et al. (2014).*

(1) $$\begin{array}{c} \left[{Ca}^{2+}\right]=[\left(F-F_{\min}\right)/\left(F_{\max}-F\right)]*K_{d}\,\;\;\;\;\;\;\;\;\;\;\;\;\;\;\;\;\;\;\;\;\;\;\;\;\;or\\ {}[\begin{array}{c} {Ca}^{2+} \end{array}]=[(R-R_{\min})/\left(R_{\max}-R\right)]*K_{d}\,\; \end{array}$$

However, if the Hill coefficient of the binding curve diverges strongly from 1 the assumptions underlying Eq.1 are violated (**Figure 1A**).

Owing to four cooperative calcium-binding sites in most GECIs, response curves frequently are highly non-linear. For example, Hill coefficients of recent GCaMP5 or six variants range from 2.5 to 4 (Akerboom et al., 2012; Chen et al., 2013), as do those of many FRET-based Cameleons (Horikawa et al., 2010; **Table 1**). Quantification of [Ca^2+^]_i_ using the linear approximation (Eq. 1) is therefore impossible (**Figure 1A**). This will affect the ability to infer calcium transients from fluorescence data. Even if the non-linearity is taken into account computationally (Akerboom et al., 2012; Lütcke et al., 2013a), the sub-linearity in the low-calcium regime of the indicator is still of particular concern. The resting calcium concentration of a cell can vary depending on cell type, cell health, and exogenous ion concentrations (Schiller et al., 1995; Helmchen et al., 1996; Maravall et al., 2000). In the sub-linear regime of the indicator these differences will lead only to minor changes in resting fluorescence. The latest generation of GCaMP type single fluorophore sensors reached their exceptional signal to noise ratio (SNR) to a large degree thanks to a combination of increasing maximum brightness at saturation and decreasing the resting brightness F_0_. Improving sensors by reducing F_0_, however, makes it more challenging to quantify differences in resting calcium because the resulting minor fluctuations in resting fluorescence can essentially not be distinguished from variations in indicator expression level.

An additional complication for the quantitative use of GECIs is that it is not clear if the non-linear relation of F or R and calcium is constant, especially considering the variable expression levels over time and between subjects. The result is that the same absolute change in calcium may lead to highly variable changes in fluorescence depending on the actual resting calcium concentration (**Figure 1A**). As a result of this variability, establishing a ‘ground truth’ of single AP-evoked fluorescence in order to infer spike rate and timing from the fluorescence data is challenging: since it is unclear from which resting calcium level single AP transients are arising, generalizing a single waveform of this unitary event to an entire population of cells can be problematic. Of course, when the indicator affinity is high enough so that the calcium changes of interest largely fall in the linear range of the indicator, reliable spike inference should be possible. Careful *in situ* calibrations of indicator fluorescence change vs. simultaneously measured cellular activity under realistic indicator expression levels and imaging conditions need to be performed in order to deduce reliable spike timings from non-linear GECI data. In these cases one should consider if more linear ratiometric GECIs would provide a better quantifiable alternative. To increase the accuracy of methods for calcium measurement and AP inference, reducing calcium-binding sites as performed with recent ratiometric “Twitch” calcium sensors (Thestrup et al., 2014) should be a design goal for other future GECI developments.

## BUFFERING AND EXPRESSION LEVEL

All calcium indicators act as calcium buffers. Therefore, expression of any type of GECI will inadvertently change the spatio-temporal dynamics of this ubiquitous secondary messenger. The degree to which an exogenous buffer affects cellular free calcium ([Ca^2+^]_i_) is well understood and largely depends on three main factors: its mobility, affinity (including binding rates), and concentration (Zhou and Neher, 1993; Neher, 1995; Helmchen et al., 1996).

If one aims at monitoring neuronal activity, i.e., calcium signals associated with APs or synaptic activation, one can either choose to minimize the effect of exogenous buffer on endogenous calcium signaling by minimizing the indicator concentration, or to maximize the SNR of the readout of calcium activity by finding the indicator concentration that yields optimal SNR.

Under ideal (i.e., photon shot noise limited) conditions, the measure of confidence that one can attribute to a change in fluorescence given the intrinsic variability in the measurement due to the Poisson statistics of light detection (i.e., the SNR), is directly proportional to the indicator’s signal change over baseline fluorescence (i.e., Δ*F* or Δ*R*) and to the square root of the baseline fluorescence signal (Yasuda et al., 2004; Göbel and Helmchen, 2007):

(2) $$SNR=\Delta F/F_{0}^{1/2}\,$$

In the case of ratiometric indicators, the relative shot noise components of donor and emission fluorescence add so that for the same relative change in fluorescence ratio from a comparable baseline fluorescence level the SNR is worse than for single fluorophore GECIs that require only one noise-affected measurement. However, since FRET indicators are typically much brighter at rest this disadvantage is largely compensated (but see Wilt et al., 2013). Increasing the concentration of a calcium indicator increases *F* and thereby improves SNR because more fluorescent molecules become available. Yet, a larger buffer concentration will also lead to a smaller fluorescence change: When the indicator is trying to bind more calcium than is entering the cell while at the same time competing with endogenous calcium buffers, the number of indicator molecules changing their emission from the baseline level decreases. The amplitude (Δ*F* orΔ*R*) and decay time constant (τ) of the calcium-dependent fluorescence change depend on the summed buffer capacity of the exogenous and endogenous buffers (Göbel and Helmchen, 2007):

(3) $$\Delta F\alpha F_{0}/\left(1+\kappa_{endo}+\kappa_{dye}\right)\,$$

(4) $$\tau \alpha 1+\kappa_{endo}+\kappa_{dye}\,\;$$

where *κ_endo_* represents the buffer capacity of the endogenous buffers (fixed or mobile) and *κ_dye_* represents the exogenous buffer capacity of the added calcium dye. The buffer capacity (or ‘binding ratio’) is the constant describing the fixed ratio between changes in free [Ca^2+^] and buffer-bound [CaB] calcium, which can be related to the effective dissociation constant (*K*_d_) and concentration [B]_tot_ of the respective buffer (Zhou and Neher, 1993; Neher, 1995):

(5) $$\left(\kappa_{dye}\right)=\Delta [caB]/\Delta {\left[{Ca}^{2+}\right]}_{i}=(K_{d}*{\left[B\right]}_{tot})/{\left(K_{d}+\left[{Ca}^{2+}\right]\right)}^{2}$$

What would be the optimal indicator concentration (or GECI expression level) to maximize SNR? *F_0_* is proportional to *κ_dye_* and by substitution in Eq. 2 one yields (Borst and Helmchen, 1998; Göbel and Helmchen, 2007):

(6) $$\begin{array}{c} S\,N\,R=\Delta \,F/F_{0}^{1/2}\,\alpha \,\kappa_{d\,y\,e}/\left[\left(1+\kappa_{e\,n\,d\,o}+\kappa_{d\,y\,e}\right)*{\kappa_{d\,y\,e}}^{1/2}\right]\\ =\kappa_{d\,y\,e}^{1/2}/\left(1+\kappa_{e\,n\,d\,o}+\kappa_{d\,y\,e}\right)\;\;\;\;\;\;\;\;\;\;\;\;\;\;\;\;\;\;\;\;\;\;\;\;\; \end{array}$$

It follows that maximal SNR is achieved under ‘balanced loading’ conditions where the endogenous and exogenous buffer capacities are equal (*κ_endo_*= *κ_dye_*; Borst and Helmchen, 1998; Göbel and Helmchen, 2007). **Figure 1B** shows this relation for various concentrations of two hypothetical GECIs at several endogenous buffer capacities that are similar to reported values for excitatory and inhibitory neurons. Also indicated are the approximated concentrations of the GECI GCaMP3 under different expression conditions. Note that for excitatory neurons (*κ_endo_*∼30–100; Helmchen et al., 1996; Maravall et al., 2000), concentrations of 25–50 μM would already yield near maximal SNR. For some classes of inhibitory neurons with high calcium binding ratio, ‘balanced loading’ would be reached at much higher expression level (e.g., *κ_endo_ ∼*285 in somatostatin positive bitufted interneurons in L2/3 of somatosensory cortex (Kaiser et al., 2001). Of course, without knowing the exact *in situ* values of all parameters (most notably the effective *K_d_*, resting calcium concentrations and *κ_endo_*), the actual optimal indicator concentration remains unknown. Complementing direct measurements of absolute indicator concentrations using purified protein as reference standard (Huber et al., 2012; Zariwala et al., 2012), the most relevant parameters for SNR-optimal ‘balanced loading’ – *κ_endo_* and *κ_dye_* – in various cell types could be experimentally obtained. By monitoring the amplitude and decay time of a step calcium signal with a calibrated second calcium indicator of different emission wavelength at different concentrations one could back-extrapolate to the apparent endogenous buffer capacity (*κ_app_ = κ_endo_*+* κ_dye_*) from the decreases in Δ*F* and τ (Eqs 3 and 4; Zhou and Neher, 1993; Neher, 1995; Helmchen et al., 1996; Matthews et al., 2013). If one would perform these experiments in cells acutely expressing the GECI (κ_app_ = κ_endo_ + κ_dye_) and compared these to control cells without the GECI (*κ_app_ = κ_endo_*) the buffer capacity added by overexpression of the GECI could be deduced and optimized. Of course, this would only be meaningful if the cell would not adjust its endogenous buffers in response to GECI expression in order to maintain buffer homeostasis – which in itself would be a highly relevant finding pointing toward potentially unwanted off-target effects of GECI expression. However, recent studies on gene expression profiling in mice globally expressing the FRET sensor TN-XXL (Direnberger et al., 2012) do not point in that direction. Nevertheless, more studies on the amount of buffering introduced into different neurons by various types of GECIs and gene transfer protocols would be highly desirable, especially when directly related to expression-correlated off-target effects of GECIs (see below). However, even with these data at hand, tailoring expression of GECIs toward SNR-optimal levels is difficult, especially when acute methods of gene transfer (e.g., viral transduction or electroporation) are used. Nevertheless, careful titration of the amount virus injected or DNA electroporated together with optimization of promoters, enhancers, or suppressors of expression should be considered to prevent unnecessary overexpression.

## INDICATOR KINETICS

It had been noticed early on that the response kinetics of GECIs were slower than that of synthetic calcium dyes. Early prototypical Cameleon-1 had a measured on-rate *k*_on_ of about 10^6^ M^-1^ s^-1^ compared to essentially diffusion-limited on-rates of 10^8^ M^-1^ s^-1^ for fura-2 or fluo-3 (Kao and Tsien, 1988; Miyawaki et al., 1997; Naraghi, 1997). Accordingly, *k*_off_ numbers were also slower, with a value of 13 s^-1^ reported for the medium affinity Cameleon-1 (Miyawaki et al., 1997). Delayed kinetics compared to synthetic dyes were subsequently confirmed for other types of GECIs based on Cam-M13 or Troponin C, albeit at varying extent (Nakai et al., 2001; Pologruto et al., 2004; Tay et al., 2007; Horikawa et al., 2010). Engineering faster GECIs has been challenging, as on- and off rates, affinities and maximal fluorescence change are tightly linked to one another (*K*_d_ = *k*_off_/*k*_on_), and it is often not feasible to optimize one parameter without losing advantageous features of the other parameters. Engineering thus mostly focused either on calcium chelating residues within the EF-hand loops or on the binding interface of calmodulin with its binding peptide. For most types of mutagenesis an expected reciprocal relationship was found between calcium affinity and kinetics (Miyawaki et al., 1997; Mank et al., 2006; Horikawa et al., 2010; Chen et al., 2013; Sun et al., 2013; Thestrup et al., 2014). Will it be possible to engineer GECIs in which both on- and off-rates are enhanced to obtain high affinity rapid kinetics sensors comparable to dyes such as OGB-1? Some mutations in the interface between Calmodulin and its binding peptide allowed to generate G-CaMP type sensors with faster kinetics, only slightly altered affinity, but smaller maximal fluorescence change (Chen et al., 2013; Sun et al., 2013). Thus, hydrophobic interactions of Calmodulin with the peptide were identified as one rate-limiting step within these sensors. Other studies on Troponin C-based FRET sensors identified slow events close to the EF-hands as rate-limiting for dissociation, while addition of the donor and acceptor GFPs had no further negative effects on kinetics (Geiger et al., 2012). More detailed structural studies on the causes of slow intramolecular dynamics within GECIs appear necessary to finally break the apparent trade-off between sensitivity and speed.

**FIGURE 2:**
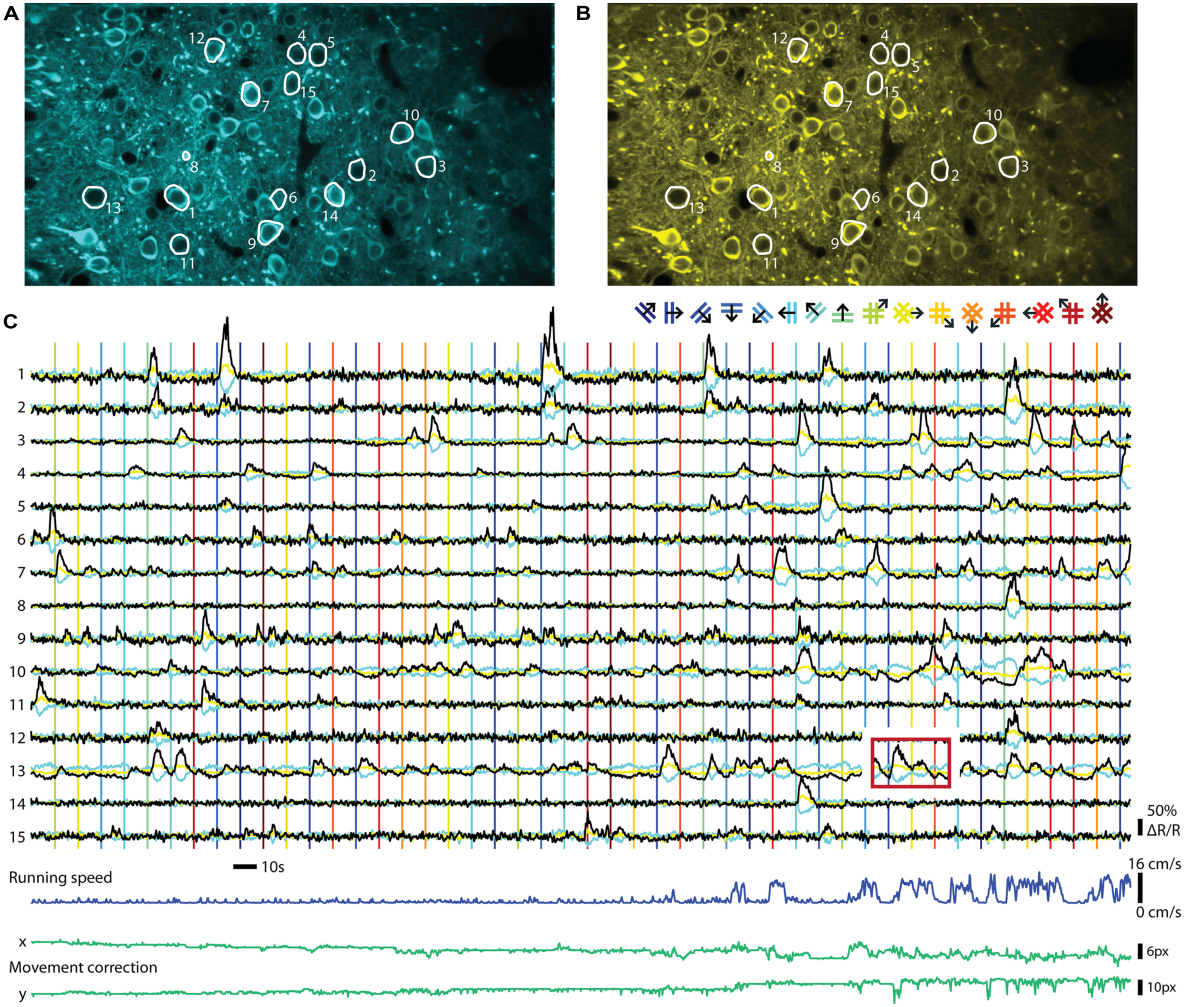
**Ratiometric imaging of neuronal activity in an awake mouse using the Twitch-2B calcium indicator.** Two-photon imaging of layer II/III excitatory neurons, conditionally expressing Twitch-2B (CAG promoter, double-floxed inverted open reading frame, 28 days after transduction by AAV1) together with Cre-recombinase (CamKII promoter, AAV1), in V1 of an awake head-restrained mouse on a treadmill (see, e.g., Keller et al., 2012). Imaging frames were acquired at 30 Hz on a custom build two-photon microscope and corrected for movement artifacts (Guizar-Sicairos et al., 2008). **(A)** Frame-averaged image of donor fluorescence (467–499 nm). Region of interest (ROI’s; white) were manually drawn for 15 active neurons. **(B)** Idem, but for acceptor fluorescence (519–549 nm). **(C)** Neuropil-subtracted (Kerlin et al., 2010) change in donor (cyan) and acceptor (yellow) fluorescence (Δ*F*/*F*) for each ROI, referenced against a 60 s moving-average. Black traces display the change in ratio of the donor and acceptor fluorescence (Δ*R*/*R*) over its 60 s moving-average. Time-series were smoothed using a 0.5 s moving average window. Vertical lines indicate onsets of visual stimuli, which were full-contrast sinusoidal moving gratings (spatial frequency: 0.045 cycle/degree; Speed: 1.5 cycle/s) or plaids (overlays of two orthogonal gratings; color of lines matches the legend above), presented on a gamma-corrected monitor at a distance of 20 cm. Stimulus presentation lasted 4.5 s, interleaved by a gray screen of equal brightness for 5.5 s. The red box over ROI 13 indicates an example of response bleed-through into the next stimulus episode. Running speed of the animal ranged from 0 to 16 cm/s (dark blue trace below). Image displacement (before correction) did not exceed 12 and 13 pixels on the x and y axes, respectively (green lines below). Data adapted from Thestrup et al. (2014).

Neurons in the mammalian CNS exhibit a wide range of firing rates, from sparse activity below 0.1 Hz (e.g., L2/3 cells in the barrel cortex, Kerr et al., 2005) to rates approaching 1 kHz (e.g., mossy fiber input to the cerebellum, Rancz et al., 2007). Even the latest generation of GECIs shows a fluorescence impulse response that rises and decays with time constants more than 100 times slower than the average inter-spike interval of the fastest neurons (**Table 1**). During high frequency activity this inevitably leads to overlapping fluorescence responses to individual APs. Once the cumulative fluorescence reaches indicator saturation level, information about the underlying neuronal activity is lost. Furthermore, experiments correlating neuronal activity with episodic sensory stimulation or behavioral events can suffer from response ‘bleed-through’: responses from previous episodes evoke activity that leads to fluorescence changes that are still present during the onset of the next episode, even though the underlying spike rate may have already decayed to baseline levels (**Figure 2C**). In these cases, raw fluorescence changes cannot be used for quantification of neuronal firing. Faster indicators lead to less response superposition and therefore allow simpler separation of individual activity events, thereby, for example, allowing shorter inter-trial intervals. However, for as long as cumulative fluorescence changes are not approaching full indicator saturation levels, the underlying spiking activity of synthetic indicators can often be resolved surprisingly well using non-linear methods of spike inference (Vogelstein et al., 2009; Grewe et al., 2010; Lütcke et al., 2013a). It remains to be established how well these methods perform with non-linear GECIs of the latest generation. The accuracy of spike inference methods depends on the actual spike rate, indicator speed, sampling rate, and all factors that affect the SNR of the measurement (see above). The complex interdependence of these factors has been extensively described elsewhere (Lütcke et al., 2013a; Wilt et al., 2013). These studies showed that faster is not always better: Depending on the frequency of expected responses, sparse sub-saturating activity will be detected with higher SNR using a slower indicator. Since imaging involves sampling at a fixed rate, more samples are collected for a fluorescence step response in the case of a slow indicator, increasing detection SNR by the square root of the number of samples acquired and preventing undersampling of short events. It is therefore non-trivial to decide – keeping all other indicator properties constant – what would be the optimal kinetics of an indicator. One needs to consider both the expected rates of activity and the sampling rate of available imaging hardware. Computational models as provided by Lütcke et al. (2013a) should ideally be consulted to make an informed decision.

## RATIOING VERSUS SINGLE CHANNEL RECORDING

The two major classes of GECIs operate in different read-out modes. While single GFP-based sensors are imaged using a single channel for recording fluorescence, FRET-based indicators are ratiometric and require splitting the emitted light into two channels that are recorded separately and the ratio of the two emission channel intensities taken as a measure of calcium concentrations. An example of a ratiometric *in vivo* recording can be seen in **Figure 2**. The indicator Twitch-2B was expressed in mouse primary visual cortex and ratiometric imaging of the activities of a group of neurons performed in awake mice. Both types of procedures have distinct advantages and disadvantages. Recording with a single channel is simpler and allows collecting all photons emitted from a probe, without any loss from emission filters or beam splitters. Such probes also occupy less bandwidth of the spectral range, allowing more multiplexing and co-labeling of neuronal cell types with different colors. Ratiometric, FRET based probes use two fluorescent proteins as fluorophores, and therefore occupy a larger area of spectral bandwidth for a given sensor. Ratioing has, however, a number of advantages if quantification of neuronal activity is desired. The ratio formed between the two channels is, in principle, independent of expression levels. Thus, heterogeneities in indicator expression levels between cells, as occur with AAV-mediated gene delivery into the brain (Aschauer et al., 2014) and other gene delivery methods, can be more easily addressed. As indicator expression levels directly affect both amplitudes and time constants of calcium signals (see, e.g., Helmchen et al., 1996), it may be necessary to correct for these differences. Direct acceptor excitation is an unambiguous way to read out indicator expression levels independent of the calcium concentration. It may also facilitate the definition of regions of interest because it will not display increased signal intensity for more active cells. During long-term imaging over months, fluctuations in excitation light intensity, changes of indicator expression levels or changes in optical path length due to tissue growth may also occur, which could be addressed by ratioing. As long noted, ratiometric imaging is beneficial when movement artifacts are a concern, such as in moving preparations or awake animals, because correlated artifacts affecting both channels in the same way are effectively canceled out due to the processing. Ratiometric imaging also corrects for further changes in optical path length resulting from vasoconstriction and -dilation. These vascular artifacts are often correlated with neuronal activity and are therefore of particular concern (Shen et al., 2012). While movement-related artifacts could also be addressed by simple co-expression of a second, preferably red fluorescent protein together with the GFP-based sensors, truly ratiometric FRET probes have the advantage that the resting ratio of the indicator can be used to directly quantify the resting calcium level. This is especially of interest for some classes of inhibitory neurons that fire APs at high rate under ‘resting’ conditions (Klausberger and Somogyi, 2008; see also Thestrup et al., 2014). Thus, the choice of indicator and read-out mode will depend on the type of experiment and the available expression systems and promoters to drive expression.

## SEGMENTATION

The main objective of calcium imaging experiments is to monitor neuronal activity via variations in fluorescence. Having performed the experiment, the next step is to make sense of the fluorescence data. Traditional methods involve hand-picking a region of interest (ROI) in the anatomy and finding the fluorescence time-series within this ROI. This method can easily be implemented when sparse labeling makes it straight forward to manually segment the anatomical ROI and is particularly useful in experiments where the experimenter knows what particular ROIs are of interest in order to answer questions such as: Is this specific neuron active in my experiment?

In certain cases though, it is more appropriate to use an automated method to select ROIs, in particular when seeking an unbiased, large-throughput way of processing the data. These methods can be said to fall roughly into two categories, those that use anatomical information and those that use functional data for the segmentation. We briefly discuss both below.

**FIGURE 3:**
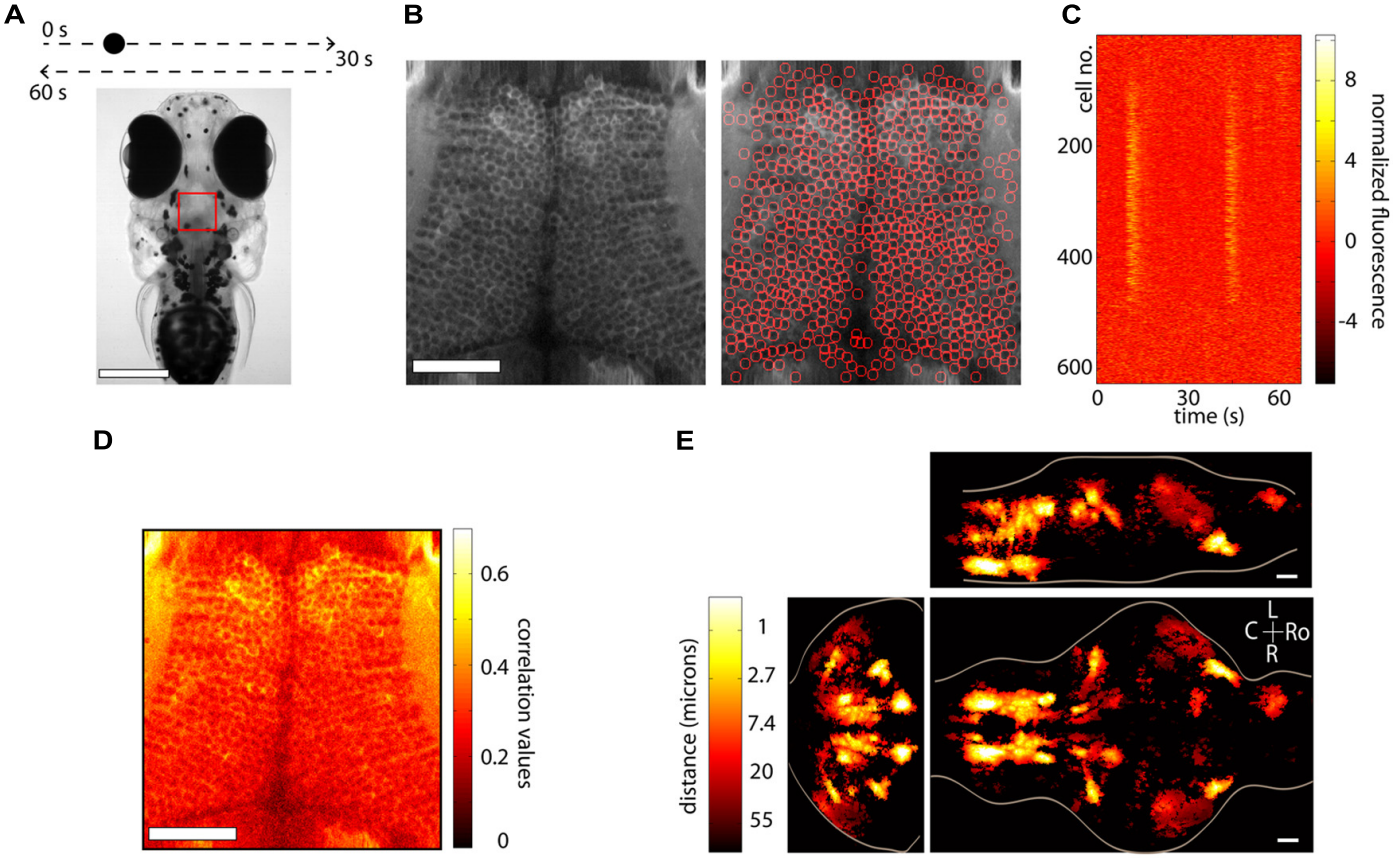
**Segmentation and whole-brain imaging. (A)** Head of a 7-day-old larval zebrafish that was embedded in agarose and was presented with a visual stimulus: a dot moving at constant speed from left to right of its visual field and then back to the left. The red square shows the area that was imaged at 7.5 Hz in the medial optic tectum. Scale bar = 250 μm. **(B)** Anatomical image of the region imaged, generated by summing all the frames acquired in one plane (left). The image can then be automatically segmented using the methods described in the text (right). In this case 627 neurons were identified. Scale bar = 50 μm. **(C)** Raster plot of the responses of the 627 automatically segmented neurons in **(B)**. **(D)** The method of computing the correlation of the fluorescence time-series of a pixel with its eight neighboring pixels is shown. This image can be used as a basis for determining functionally active cells by determining a threshold (following a shuﬄing-control) followed by segmentation. **(E)** How similar is activity during behavior across different animals? This question was addressed by imaging the whole brain of 13 behaving larval zebrafish discretized in over 500 × 800 × 400 voxels and then morphing the brains onto a reference brain (Portugues et al., 2014). Functionally active units were segmented using correlation-based methods described in the text. For every active voxel, how far on average must one look in other brains to find a similarly active voxel, i.e., one displaying similar activity patterns? The figure shows that in regions such as the ventral hindbrain neuropil, the cerebellum and certain retinal ganglion cell arborization fields, the answer is surprisingly less than 1 μm. Ro, rostral; L, left; R, right; C, caudal; scale bar = 50 μm.

Anatomical segmentation methods greatly rely on the specimen being imaged and the labeling of the tissue. Issues such as whether the calcium indicator is expressed in the nucleus or the cytoplasm, whether the labeling is dense or sparse and whether neurons are morphologically similar or vary widely in shape and size all enter the design of the particular algorithm. In regions where the morphology of the anatomy is homogenous, algorithms can be quite effective. **Figure 3A** shows an example from the optic tectum of a larval zebrafish expressing GCaMP5G under the pan-neuronal promoter *elavl3*. The neurons have their cytoplasm labeled, are densely packed and of similar size. In order to perform automated segmentation, the anatomical image is spatially filtered with a filter whose width is in the order of the diameter d of a typical neuron, for example a Gaussian filter with standard deviation d. This removes local spatial inhomogeneities and emphasizes the important features that will be used for segmentation. In this case these are the bright cytoplasms, which can be used to identify boundaries between cells and the dark nuclei which can be used to identify the centers of the cells (**Figure 3B**, left). One may then perform a watershed algorithm on this image that will identify the “ridges” in this image, namely the bright cytoplasms. This will segment the image into ROIs, many of which will be individual cells (**Figure 3B**, right). By placing constraints on the morphology of these ROIs, such as a lower and upper limit on their size it is possible to ensure that most of the ROIs that are kept are actual neurons. The fluorescence time-series for all the ROIs can then be extracted using these ROIs as masks. **Figure 3C** shows this process for the 627 automatically segmented neurons in **Figure 3B**. These algorithms are not perfect. As can be observed in **Figure 3B**, right, they will fail to identify *bona-fide* neurons and will identify ROIs that are not actual neurons (akin to type II and type I statistical errors, respectively). By placing further constraints, errors can be minimized, but simple algorithms like the ones described are able to correctly identify a large fraction of neurons within seconds. These methods have been used (Akerboom et al., 2012; Panier et al., 2013) to identify 1000s of neurons in the brain of larval zebrafish. The expression of calcium indicators can also be restricted to the nucleus using a nuclear localization sequence (NLS). This will remove labeling in neuropil and will generally make it easier to segment the signals, simply by identifying particles in the anatomical image (Prevedel et al., 2014). Alternatively, adding a spectrally separated nuclear co-label, for instance by co-expression of a red protein, would help greatly with morphological segmentation for the same reasons.

A second class of algorithms involves functional segmentation. This idea relies on the fact that pixels that belong to the same neuron will have highly correlated fluorescence time-series. Naturally, if a neuron is not active, the time-series of the pixels that comprise it will involve mainly independent noisy fluctuations that will exhibit low correlation. These algorithms will therefore identify contiguous regions that are active in a correlated way. Explicitly the algorithms work as follows. For every pixel one can compute the correlation of its time-series with the sum of the time-series of its eight closest neighbors (in the case of 2D segmentation). This can be repeated for every pixel in the image, such that the result is an anatomical image of correlation values. In **Figure 3D** we perform this analysis for the same dataset as **Figures 3B,C**. This image can then be further processed in one of two ways (potentially following spatial filtering). The easiest way is to perform a threshold operation (set a threshold and set to 0 all the pixels with values below the threshold) and then identify particles within the thresholded image. In this case the threshold can be set either by hand, or more rigorously, by performing a shuﬄing control, comparing the distributions of the un-shuﬄed and the shuﬄed correlations and using the correlation value that implements a certain confidence interval of choice (i.e., this correlation value is 20 times more common in the un-shuﬄed versus the shuﬄed data).

Alternatively, the correlation image can be used to determine the seeds of a region-growing algorithm. The first step is to look for local maxima in the correlation image. The highest maximum is then used as a seed of the first ROI, and neighboring pixels are added to the ROI if their correlation with the already existing pixels in the ROI exceeds a threshold, which should ideally be determined by again performing a shuﬄed control. This process is repeated until no more pixels are aggregated and then one proceeds to the second highest maximum, which becomes the seed of the second ROI. It is once more possible to place constraints on the size and shape of these ROIs to ensure that certain requirements are met, for example, that they have the morphology of neurons. This method will only produce *active* ROIs, as opposed to the anatomical segmentation mentioned before, and has been recently used in (Portugues et al., 2014) to automatically identify 3D ROIs throughout the brains or larval zebrafish.

In the case of the dataset shown in **Figures 3A–D**, this method would not work particularly well to identify individual neurons, because many contiguous neurons are active and would be clumped into the same ROI. On the other hand, using this algorithm will identify activity in regions which are not morphologically different from their anatomical surrounding, such as neuropil.

No single method is superior to the others and which one should be implemented depends on many factors, such as the biological questions that need to be answered, the specific expression pattern of the indicator or the signal to noise of the measurement. These are by no means the only algorithms possible. Functional segmentation can be performed using maximum DF/F instead of correlation with neighboring pixels as a measure of activity and then centering ROIs that are the size and shape of typical neurons on the spatial locations of maxima that exceed a threshold (Ahrens et al., 2012), methods involving independent component analysis have been developed (Mukamel et al., 2009), and of course it is always possible to mix and match.

## CHRONIC IMAGING

Neuronal circuits adapt in response to sensory experience, mature during development and change due to disease processes in time scales which vary from milliseconds to months. While fast events are easily captured with electrophysiology techniques and imaging of synthetic calcium dyes, events that extended more than a few hours in time were up to now hard or impossible to follow due to technical limitations. Long term imaging of structural changes within the nervous system (see e.g., Grutzendler et al., 2002; Trachtenberg et al., 2002) have been performed routinely using anatomic labeling with fluorescent protein variants, but these studies lacked a physiology component. GECIs were soon identified as a suitable means to follow activity of identified groups of neurons in repeated sessions over long periods (for review see Aramuni and Griesbeck, 2013; Lütcke et al., 2013b). After the first demonstration of chronic imaging of sensory induced activity in mouse visual cortex over weeks (Mank et al., 2008) a number of studies have extended the paradigm to other brain areas and increased the intervals between imaging sessions up to a month or longer (Tian et al., 2009; Andermann et al., 2010; Dombeck et al., 2010; Minderer et al., 2012). By now, numerous high-end applications reveal new insights into reconfiguration of network properties as a consequence of learning and plasticity. Huber et al. (2012) followed populations of neurons in motor cortex over weeks while mice learned a new object detection task. They reported strengthening of the task representation at the level of the population, which was stable despite of the dynamics at the single neuron level. In another study (Margolis et al., 2012) populations of neurons in barrel cortex were followed over time after sensory deprivation, demonstrating the power of chronic long term calcium imaging to monitor response dynamics within individual neurons and across whole populations of neurons as they undergo plasticity. Exciting recent papers describe re-organization of population activity in motor cortex after standardized learning of a somatosensory task (Masamizu et al., 2014). With the advent of GECIs with exquisite sensitivities and established biocompatibility it is expected that a plethora of new studies on long term physiology will provide new insights into long standing questions, e.g., on how the brain manages to balance between stable representations and adaptation due to sensory experience, on how it couples sensory input to behavioral output, on how it fine-tunes circuitry during development, how it uses population coding to represent, store and retrieve information of the outside world, and finally also on how pathologic change and circuit dysfunction in the brain is causally manifested.

## WHOLE-BRAIN IMAGING

The dream of a systems’ neuroscientist is to be able to record the spiking activity (the membrane potential would even be more preferable) of all the neurons in a brain while the animal is actively engaged in a behavior. Recent studies now show that this is a very real possibility, at least in certain model organisms.

The nematode *C. elegans* and the larval zebrafish are transparent organisms, small enough so that a large fraction of their nervous system fits within the field of view of an objective. They have cells that on average range from 3 to 10 microns in diameter, although *C. elegans* have 302 and zebrafish in the order of 150,000. Traditional approaches involving point scanning microscopy required several presentations of the same experimental paradigm per plane in order to determine the calcium response properties of the cells in the imaging plane. However, the signal to noise properties of the latest GECIs (Akerboom et al., 2012; Chen et al., 2013; Thestrup et al., 2014) allows the unambiguous determination of neuronal activity from single trials. This reduces the imaging time by three- to ten-fold, allowing the imaging of a whole brain in the order of 4–10 h, with x, y, and z resolution of ∼1 micron. When dealing with robust behaviors, such as the optokinetic reflex, this can be used to obtain functional maps with single-cell resolution of neuronal activity throughout a single brain and create whole-brain anatomical maps of both stimulus and motor related activity (Portugues et al., 2014). In many instances the neuronal activity shows correlation values with these variables exceeding 0.7. These neuronal networks are sparse, with around 5% of the brain showing consistent activity, yet highly stereotyped across individuals, often within the extent of a single cell body (**Figure 3E**). These experiments revealed some more surprising features, such as spatial gradients of temporal activation along neuropil regions in both the hindbrain and retinal ganglion cell arborization fields, highly asymmetric activity mostly in the left habenula, and a small number of individual cells in the optic tectum which, despite receiving direct retinal input only from the contralateral eye, displayed binocular responses.

Scanning microscopy nevertheless has its limitations. The activity during single trial learning, for example, cannot be observed in every imaging plane. The nuclear targeting of calcium indicators and the implementation of more recently developed volumetric imaging techniques such as fast z-scanning with piezos (Göbel et al., 2007), electrically tunable lenses (Grewe et al., 2011), acousto-optic deflectors (Grewe et al., 2010), light-sheet imaging (Ahrens et al., 2013; Panier et al., 2013), temporal focusing of sculpted light (Schrödel et al., 2013), and light-field imaging (Prevedel et al., 2014) have opened the door to the possibility of monitoring whole-brain activity with an improved temporal resolution: 1 brain every 0.5 s as opposed to every several hours, at the expense of slightly reduced spatial resolution. Although certain technical difficulties still need to be overcome, it is clear that the calcium indicators now available are contributing to turn the dream into a reality.

## OFF-TARGET EFFECTS OF GECIs

**FIGURE 4:**
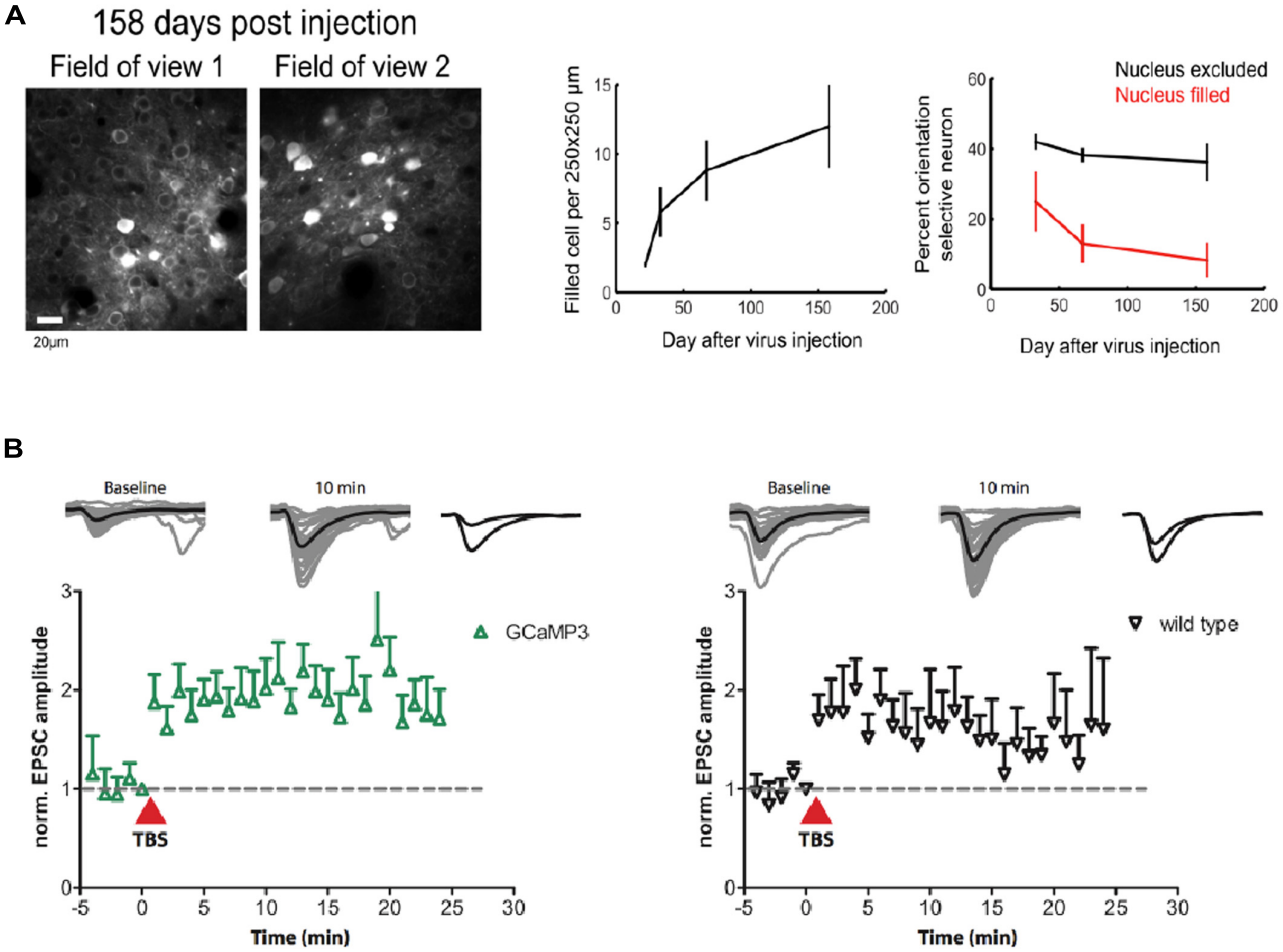
**Biocompatibility and off-target effects of GECIs. (A)** Long-term high-level expression of a GECI (GCaMP6s) leads to breakdown of nuclear exclusion of the indicator. The number of ‘filled’ cells is a function of time after viral transduction (middle panel). Cells with ‘filled’ nucleus show atypical functional responses (right panel): neurons in primary visual cortex with ‘filled’ nuclei lose their orientation selectivity in response to moving grating stimulation. **(B)** GCaMP3 expression at moderate levels (∼15 μM) in CA1 neurons in rat hippocampal slice culture does not affect the early phase of long-term potentiation (LTP) in the short run. [Figures reproduced from Huber et al. (2012) and Chen et al. (2013) with permission (pending)].

A major problem for previous GECIs has been that SNR-optimal indicator concentrations often could not be reached without leading to obvious signs of deterioration of cell health or compromises on indicator function. Indeed, already early versions of Cameleons showed sensor inactivation or formation of aggregates when expressed in transgenic mice (Hasan et al., 2004; Nagai et al., 2004). Contemporary viral or non-viral expression methods (e.g., *in utero* electroporation) lead to high and often steadily increasing chronic expression levels. By comparing the fluorescence of purified protein with the fluorescence of GECI-expressing cells, the actual indicator concentration has been determined under various conditions for GCaMP2 and GCaMP3 (Hires et al., 2008; Huber et al., 2012; Zariwala et al., 2012). Expression levels that are likely to exceed SNR-optimal concentrations have been achieved after 7 weeks of viral transduction of cortical neurons with GCaMP3 (Huber et al., 2012; **Figure 1B**). This high expression level led to breakdown of nuclear exclusion of the indicator in a considerable number of cells, a phenomenon that is known to correlate with clear signs of changed cellular response properties. A variable degree of nuclear filling has been observed for essentially all indicator classes, with the Troponin-based TN-XXL showing the smallest tendency for nuclear accumulation (Tian et al., 2009). Also in the case of GCaMP5 and GCaMP6, long-term high-level expression led to a large number of cells with affected nuclear exclusion and atypical cellular responsiveness (Tian et al., 2009; Akerboom et al., 2012; Chen et al., 2013). Overexpression using AAVs at the viral titers available at public virus repositories led to a steadily increasing fraction of ‘filled’ cells in mouse visual cortex that correlated with aberrant cellular tuning responses (**Figure 4A**). Further comparative research into the biocompatibility of various GECIs at different expression levels is clearly warranted.

Either by means of changed expression strategies or serendipitous improvement of indicator properties, general cytotoxicity of the GCaMP family of indicators appears to have been reduced over time. Constitutive expression of GCaMP2 in mice led to unwanted phenotypes like cardiac hypertrophy (Tallini et al., 2006), whereas similar effects have not been noted for later conditional or neuron-specific GCaMP2, GCaMP3, and GCaMP5 transgenic mouse lines (Chen et al., 2012; Zariwala et al., 2012; Gee et al., 2014). While GCaMPs were readily expressed in transgenic flies and fish, currently available mouse lines for GCaMP3, however, still suffer from relatively low expression levels, that so far have prevented their widespread use in the community. Early versions of Troponin C based sensors could be expressed functionally and stably in transgenic mice at high levels (Heim et al., 2007), but their lower signal strength made them unsuitable for a number of high end applications. Healthy animal models constitutively or conditionally expressing an SNR-optimal concentration of a high performance GECI would be desirable. The first generation of publicly available mouse lines expressing GCaMP6 (GCaMP6s, GCaMP6f under the Thy1-promoter) has just been released (Dana et al., 2014). GECIs now have reached a state of maturity that makes waiting for the ‘next-best’ version less tempting. Given suitable expression levels and patterns, we expect that mouse lines like these will become widely popular in the future.

Stably or conditionally expressing animal models greatly simplify imaging by rendering invasive acute transfection methods unnecessary and by improving the repeatability of experiments. Probably even more importantly, they ameliorate issues resulting from the unavoidable ramping up of expression after viral transduction that render long-term chronic imaging of the same cell-populations problematic. Chronic imaging of stably expressing neuronal populations is indispensable, though, to study experience-dependent plasticity on the single cell level. Changes in intracellular calcium determine the sign and amplitude of synaptic plasticity (Shouval et al., 2002). GECI overexpression has therefore always been suspected to affect neuronal plasticity. While plastic changes during learning have now repeatedly been observed using chronic GECI expression over weeks (Huber et al., 2012; Masamizu et al., 2014) and months (Margolis et al., 2012), the observed experience-dependent changes could in principle also have occurred in associated, untransfected upstream circuits. However, experiments on hippocampal slice culture that expressed GCaMP3 at moderate levels have shown that early phase long-term potentiation (LTP) is indistinguishable from non-expressing control (Huber et al., 2012; **Figure 4B**). Still it is necessary that further experiments compare the achievable levels of *in vitro* synaptic plasticity between expressing and non-expressing cells in brain slices from the very animals, brain regions, cell types, and expression levels used for the chronic experiments – ideally accompanied by estimates of added buffer capacity and put in relation to adverse phenomena like nuclear filling.

## REASONS FOR RED

The most recent optimization efforts in the field of GECI engineering are centered on expanding the spectral palette of high-performance GECIs. While indicators emitting in the blue range of the visible spectrum have been developed as well (Zhao et al., 2011), most work so far focused on the development of viable probes emitting in the red (Ohkura et al., 2012b; Akerboom et al., 2013; Wu et al., 2013). The reason for this is that red indicators would have desirable properties that go beyond the most obvious advantage of being able to spectrally multiplex different cellular populations expressing GECIs of different color. Current generation GECIs have their single photon excitation maximum around 440–480 nm. This, however, poses a problem if one tries to use these optogenetic sensors together with optogenetic actuators like Channelrhodopsin-2 (ChR2; Akerboom et al., 2013; Wu et al., 2013). While two-photon laser scanning microscopy can be used to largely prevent co-excitation of ChR2 (Zhang and Oertner, 2006), single photon activation of ChR2 inadvertently strongly excites most GECIs due to excitation spectral overlap. This not only affects the functional fluorescence readout but may also lead to unnecessary photobleaching and damage. Red proteins usually require green excitation light (550–560 nm), which renders cross-excitation less critical but not entirely unproblematic: ChR2 can still be effectively excited by intense green light given suitable expression levels (Zhang and Oertner, 2006). A further benefit of red GECIs would be that they would allow imaging deeper with less excitation power. Light scattering is strongly wavelength-dependent. The shorter the wavelength, the higher the probability of photons straying off-course from their ballistic path. As a very coarse approximation, both excitation and emission light (in both the single and two-photon excitation regime) could travel roughly twice as far without being scattered due to refractive index mismatches in brain tissue if excitation and emission light would be shifted 100 nm to the red in comparison to commonly used ‘green’ probes (Helmchen and Denk, 2005). Further red-shifted probes may even allow for near infrared intravital imaging of Ca^2+^ signals through skin and bone. In addition to increased depth penetration, red-shifted excitation in both single- and two-photon imaging modes leads to a reduction in the background signal resulting from the excitation of autofluorescence, thereby increasing SNR. If the overall GECI fluorescence is low, activity-dependent changes in autofluorescence (e.g., of flavoprotein oxidation) can become a major source of signal contamination. Intrinsic changes in flavoprotein fluorescence are widely used to map cortical responses to sensory stimuli (Shibuki et al., 2003; Michael et al., 2014). The sign of the signal change as well as excitation and emission wavelengths of flavoprotein autofluorescence overlap with those of ‘green’ GECIs. Red GECIs would therefore be especially helpful for wide-field single photon imaging where the source of the fluorescent signal cannot be confirmed spatially. So far, however, red single fluorophore GECIs like R-GECO and RCaMP have not reached the same performance as their green counterparts and still suffer from low SNR, strong photobleaching and even photoswitching artifacts that have so far prevented their wide-spread use (Yamada and Mikoshiba, 2012; Akerboom et al., 2013; Wu et al., 2013). An alternative might be red-shifted FRET indicators based on new engineered bright green or yellow (as donors) and orange or red fluorescent proteins (as acceptors; Tsutsui et al., 2008; Lam et al., 2012; Shaner et al., 2013). Incorporation into current designs such as “Twitch” indicators will require some substantial re-engineering of the indicators, but further increases in brightness, a better separation of donor and acceptor emission channels and the overall red-shift promise a significant boost in sensitivity of these sensors.

## CONCLUSION

Genetically encoded calcium indicators have come a long way since the presentations of the initial designs. Cycles of iterative improvements, biophysical, and structural analysis and testing have led to variants with ever increasing signal strength. Recent engineering efforts have also aimed at both lowering calcium buffering by the sensors and improving linearity of responses. Finally, large-scale mutagenesis and screening approaches have resulted in high performance variants in both FRET-based and GCaMP indicator families. In particular, these latter efforts provide a viable example for improving some of the other genetically encoded sensors that neuroscience is interested in, for example sensors of membrane potential. With the remaining issues clarified, as pointed out in this article, imaging of GECIs will finally become a tremendously valuable and mature set of tools for analyzing neuronal circuits and their plasticity and pathology.

## Conflict of Interest Statement

The authors declare that the research was conducted in the absence of any commercial or financial relationships that could be construed as a potential conflict of interest.
